# Simplified Testing of the Bond Strength of Adhesives Used for Bonded Anchors

**DOI:** 10.3390/ma14123298

**Published:** 2021-06-15

**Authors:** Jan Barnat, Jan Prokeš, Miroslav Bajer, Ondřej Bezděk, Martin Vild

**Affiliations:** 1 Faculty of Civil Engineering, Brno University of Technology, 602 00 Brno, Czech Republic; bajer.m@fce.vutbr.cz (M.B.); vild.m@fce.vutbr.cz (M.V.); 2 PREFA KOMPOZITY, a.s., 615 00 Brno, Czech Republic; prokes@prefa.cz (J.P.); bezdek@prefa.cz (O.B.)

**Keywords:** bonded anchor, bond stress, tension force, adhesive, fillers, experiment

## Abstract

The analysis presented in this paper is focused on problems of bond strength as an overall bond quality parameter of industrial adhesives for structural anchoring. In the first part, the problem of bond strength as the most important parameter influencing the final anchor resistance to tension load is described. Further in the text, a new methodology of simplified testing of the strength parameters of adhesives is described. Special test specimens made from steel are repeatedly used in this methodology. Additionally, results of these tests on some new recipes for adhesive are presented. Especially, epoxy resins with special fillers, such as carbon fibres, carbon nanotubes or graphene, were tested. The use of these adhesives in temperatures close to zero degrees Celsius was also tested.

## 1. Introduction

The anchoring of building structures by the use of post-installed chemical anchors remains one of the most interesting topics for many researchers in the world for the last several decades. The reason for this is that there still are some actual problems in the field of production, design and use of such anchoring systems. There are many research studies dealing with the problem of the determination and description of the bonded anchor load-bearing capacity using experimental methods [1,2] or numerical simulations [3,4,5]. This problem is quite complex because this anchoring system usually consists of several materials (anchor bolt material, structure material in which the anchor bolt is installed and the contact material providing the connection).

The load-bearing capacity can be limited by the strength or strain characteristics of each one of these materials or by the combination of more than one of them. Therefore, the resistance of the bonded anchor is also usually determined for several types of failure depending on the load character and direction. Many authors have suggested relationships derived from the results of experiments and numerical studies, and there are relationships based purely on the mathematical regressions of the results, without a direct link to specific material characteristics [6].

The most discussed types of failure are located in the zone of basic material, either as a failure of the basic material itself or as an interaction between the basic material and the contact material (adhesive). The basic material can differ; the ones mainly used are concrete, masonry or wood. This paper is further focused especially on bonded anchor installed into a concrete base and loaded by static tension force. The resistance of the bonded anchor in this configuration depends on the concrete and glue characteristics from the form of the viewpoint of the material used.

The quality of the connection of the steel anchor bolt mediated by the adhesive is standardly verified by a tensile test performed in concrete specimens [7]. As explained below, concrete does not always affect the result of such a test. The main novel contribution of this article is the use of a new, simplified method of testing the quality of the adhesives in their development. The method consists of the use of a steel base specimen, which can be cleaned repeatedly and is particularly suitable for the continuous testing of developed adhesives. It saves time, space and material and provides sufficiently accurate information about the main parameters affecting the resulting quality of the adhesive.

This method was used in the development of new adhesives for specific conditions. Mainly, the variations of epoxy resins, for use in combination with high-strength concrete, were tested. In this study, the effect of different filler types on the behaviour of the epoxy resin was tested. In addition, various curing accelerators have been tested to improve the applicability of the adhesive at low temperatures. The result of this analysis is a proposition of the new adhesive compound that adequately takes into account both the requirements of high-strength and low-temperature applicability.

### 1.1. Motivation

The design of the adhesive anchor has to be economical and effective. From that point of view, considering all parameters influencing the load-bearing capacity, the materials and the geometry have to be well chosen. In cases where the main parameters influencing the strength of the anchor are concrete or steel, it is relatively easy to choose a better grade of steel for the anchor itself or a better grade of concrete in order to increase the strength.

However, there are also configurations in which the quality of the bond between the steel and the concrete that is mediated by the adhesive may be limiting. These situations in particular include, unfavourable conditions during the installation of the anchor, especially low temperature [8], or a specific geometric configuration, such as a small anchor length combined with high concrete strength [9]. The mechanical properties of the adhesive itself play a major role in both of the cases above. The results of the research, which are discussed in more detail below, were therefore focused on the possibility of increasing the strength of the adhesives and, at the same time, also on the methodology and possibilities of a simplified and faster verification of the properties of new adhesive recipes.

### 1.2. Failure Types

The possible failure of the adhesive anchor loaded by static tension force can occur in several ways. In uncracked concrete, the main failure types of the single anchor are steel failure, pull-out failure (bond failure), concrete cone failure and concrete splitting failure, which occur when the anchor is installed near the edge of the concrete body [1,10,11,12,13].

#### 1.2.1. Concrete Cone Failure

The relation (1) can express the tension resistance of the adhesive anchor in the case of the concrete failure mode. It is based on the CCD—concrete capacity design method according to [14]. The coefficient *k* changes in dependence on the anchor type or other conditions. In the design standards [7,15,16], the value *k* varies in range from 7.2 to 11 for adhesive anchors, in dependence on the concrete type. This failure does not depend on the bond strength provided by the adhesive.

(1) $$N_{u}=k\cdot h_{ef}^{1.5}\cdot \sqrt{f_{c,k}}$$

where *h_ef_* is the embedded length of anchor and *f_c,k_* is the cubic compressive strength of the concrete.

#### 1.2.2. Bond Failure

Bond failure is the failure of anchor extraction from the concrete body without significant concrete failure. Two bond stress models can express the relation describing the tension resistance for this type of failure. The first one is the uniform bond stress model (2). The tension resistance of the anchor in this model is defined by the embedment length, the diameter of the drilled hole and the uniformly distributed shear stress τ on the corresponding surface.

(2) $$N_{u}=\pi \cdot \tau \cdot h_{ef}\cdot d_{0}$$

where the *τ* is the uniformly distributed shear stress on the surface defined by the embedment length *h_ef_*, and the diameter of the drilled hole is *d*_0_.

The second elastic bond stress model (3) [5,17] gives similar results as the uniform bond stress model, but it is more reliable in the case of longer embedded lengths [18].

(3) $$N_{u}=\pi \cdot \tau_{max}\cdot h_{ef}\cdot d_{0}\cdot \left(\frac{\sqrt{d_{0}}}{\lambda^{\prime }}\cdot \tanh\frac{\lambda^{\prime }\cdot h_{ef}}{\sqrt{d_{0}}}\right)$$

where *τ_max_* is the peak value of shear stress near the top edge of the anchor bolt, and the value *λ’* is the elastic constant, which depends on the shear stiffness of the adhesive–concrete system and the axial stiffness of the anchor. The bond stress model is more often defined for the interface between the steel anchor bolt and the adhesive (mortar) using the diameter *d* of the anchor bolt in design standards [7,15,16] such as (4). The bond stress, in this case, has to be defined on the same interface.

(4) $$N_{u}=\pi \cdot \tau \cdot h_{ef}\cdot d$$

#### 1.2.3. Combined Concrete–Bond Failure

The relations (1), (2), (3) and (4) are highly reliable in cases when mainly one of the materials affects the failure (concrete or the adhesive). In many cases, the failure is the combination of concrete and bond failure. In that case, the tensile anchor resistance can be expressed as a combination of previously introduced relations according to [5,17]. For the uniform stress model, an example is (5).

(5) $$N_{u}=0.92\cdot h_{ef}^{2}\cdot \sqrt{f_{c,k}}+\pi \cdot \tau \cdot \left(h_{ef}-\frac{\pi \cdot \tau \cdot d_{0}}{1.84\cdot f_{c,k}}\right)$$

Another relation [19], derived from experimental results, combines the concrete and bond failure as (6).

(6) $$N_{u}=0.74\cdot \pi \cdot \tau \cdot h_{ef}\cdot d_{0}\cdot \left(1-e^{-1.5\left(\frac{f_{c,k}}{\tau }\right)}\right)$$

Except for the concrete cone failure described by relation (1), all other models are using the value of the shear stress on the contact interface. This value depends mainly on the adhesive characteristics, and it has to be assessed by experiments. The last failure type is the rupture of the steel anchor bolt. The cross-section area and the material of the anchor bolt define this failure.

### 1.3. Factors Affecting Adhesive Anchors

The failures of adhesive anchors are in general influenced by many factors, which can be marked as installation factors, service factors or factors directly influenced by material characteristics [1,10,11,12,20,21,22,23]. The main installation factors are mainly: anchor diameter, embedment depth, the drilling procedure and type of the drill, installation temperature, moisture condition and the drilled hole’s orientation. The geometric parameters of the anchor have a direct effect on its load-bearing capacity. Especially for the concrete cone failure or a combined concrete–bond failure, in addition to the direct effect of geometry on stress, it is possible to discuss the effect of a deterministic size effect. Crack propagation in concrete can be affected by the size of the stressed area of concrete around the anchor, which is directly determined by the diameter of the anchor bolt, the embedment length, the thickness of the adhesive layer and the size of the concrete body. However, according to [7,10,16,17,18,19] the mentioned relations (1), (2), (3), (4), (5) and (6) are valid only in a limited range of geometrical parameters which were experimentally tested. This range is also related to the common dimensions of anchors used in practice. For the diameter of the anchor bolt *d*, it is the range from 10 to 50 mm, for the anchoring depths, in the range 4*d* to 20*d*. These results do not show a significant influence of deterministic size effect on the results.

The main service factors are mainly: variable service temperature, possible exposure to extreme temperatures, the number of the freeze–thaw cycles, moisture conditions and possible exposure to chemical and physical hazards.

The maximum service temperature is mainly determined by the glass transition temperature of the resin. It is generally known that the maximum service temperature should be about 10–20 °C lower than the glass transition temperature. Temperature-dependent load-bearing capacity and stiffness of chemical anchors are characterised in the literature. For the temperatures from room temperature to 130 °C, this phenomenon is very well described, for example, in [24].

The main factors related to the used materials are: strength, age and the type of the concrete, initial and the final strength of the adhesive, type and strength of the steel of the anchor bolt and the surface treatment of the steel anchor bolt.

Some of these factors influence the final resistance of the anchor directly. Those characteristics (especially material and the geometrical installation factors) are usually the direct inputs into the relations describing the resistance of anchor for the specific failure type. The other factors, mainly the service factors and some installation factors, are in common design methods covered by safety factors or their effect is eliminated or reduced by the manufacturer’s terms of use [7,16].

From the point of view of analysing the bond strength provided by the adhesive, which is the main topic of this article, the most important parameters influencing the tension resistance of the anchor are the factors influencing the interfaces between the steel anchor bolt, adhesive and the concrete as well as the factors influencing the curing process of the adhesive. The most important factors are the type of the drilling, moisture conditions, the process of cleaning the drilled hole and anchor bolt and the temperature during the installation of the adhesive and during the curing process.

The effect of the cleaning procedure on the final bond strength can be significant. The standard cleaning process is a combination of brushing the drilled hole with the steel brush followed by the removal of dust by air-blowing. According to [8], the reduction of the bond strength can be in the range of 20–60% when the brushing process is omitted. A reduction of 60% was also observed for the anchor in an uncleaned hole.

The hole-drilling method has a similarly significant effect. The standard recommended method is drilling with a hammer drill. It is one of the most suitable methods. The test results published in [12] show slightly better results (10%) achieved by the use of compressed air drilling. The use of a diamond drill, which creates a surface that is too smooth in the hole, seems unsuitable; the resulting bond strength can be reduced by up to 80% compared to a hammer drill.

### 1.4. Bond Strength and Failure Mechanisms

**Figure 1:**
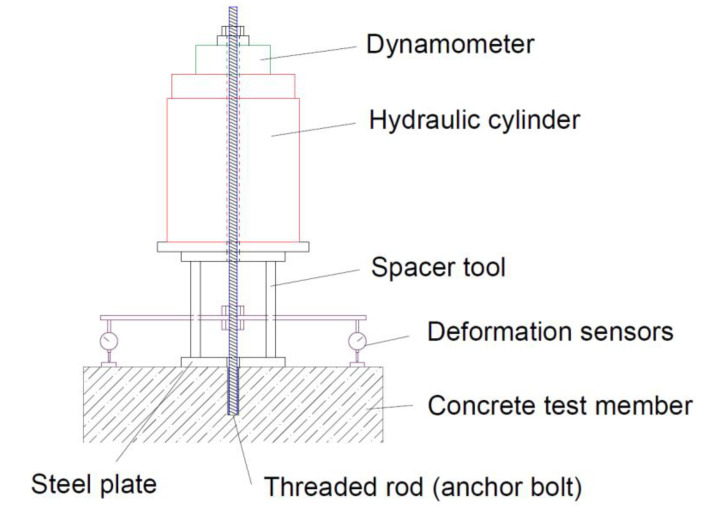
The principle of the confined test of bond strength. It is the similar configuration as for confined test according to the standard ETAG 001 [7].

Bond strength is a value obtained by the so-called confined test [7]. In principle, it is a pull-out test of an anchor where the concrete failure is prevented by the loading system itself; Figure 1.

**Figure 2:**
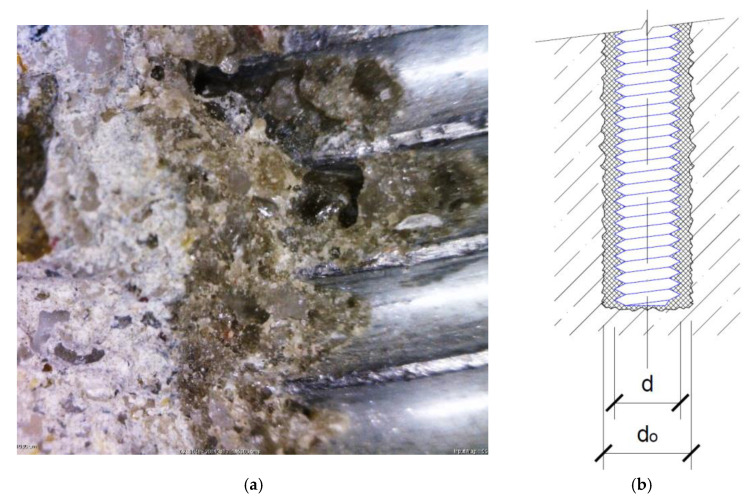
(**a**) Microscope photo of bonded anchor—contacts between materials, from the left-hand side—concrete/adhesive/steel anchor bolt; (**b**) Interface concrete–adhesive defined by outer diameter of drilled hole *d*_0_ and interface adhesive–anchor defined by outer diameter of the anchor bolt *d*.

The value of the bond strength is, in fact, the peak value of tangential stress measured on the contact area. This contact area is given by the anchor bolt diameter and the anchorage length. In ETAG 001 [7], the diameter of the anchor bolt itself is used. However, the real failure occurs on the interface between the steel and the adhesive only when the adhesive is the material with the lowest strength characteristic [19]. In common-strength concrete, failure usually occurs on the interface between the adhesive and the concrete. The thickness of the adhesive layer for the most widespread adhesive anchor systems is usually between 1 to 2 mm. This means that in common cases, it is acceptable to simplify the solution this way. As it is shown in Figure 2a,b, the surface of the concrete after drilling is quite rough (considering the use of a hammer drill).

This implies that failure on this contact is not the problem of pure adhesion. Thanks to the articulation of the contact and the interconnection of materials, there are local shear-stressed surfaces on the interface. The final contact failure is determined mainly by the shear strength of the individual materials. Depending on what concrete is used and what adhesive is used, each of these materials contributes to the failure to a different extent. This fact can lead to a combined failure of the concrete and the adhesive [17,18].

#### Failure Mechanism and Post-Peak Behaviour

Both the unconfined (pull-out test of the anchor) and the unconfined test (bond strength tests) defined by the ETAG standard [7], as well as the presented simplified method of adhesive testing, all use load-controlled testing. None of these methods is intended to describe the behaviour of the contact after reaching the maximum load capacity in cases when the loading is controlled by the rising force. However, the description of the post-peak behaviour is important to understand the failure mechanism. For the possibility to observe the post-peak behaviour the pull-out tests have to be performed using the deformation-controlled loading system. FEM numerical models also often solve the problem of the post-peak behaviour of the anchor. For the anchor loaded by tensile force, for both the confined and unconfined test, well defined model in the comparison to experiment results were published in [25].

**Figure 3:**
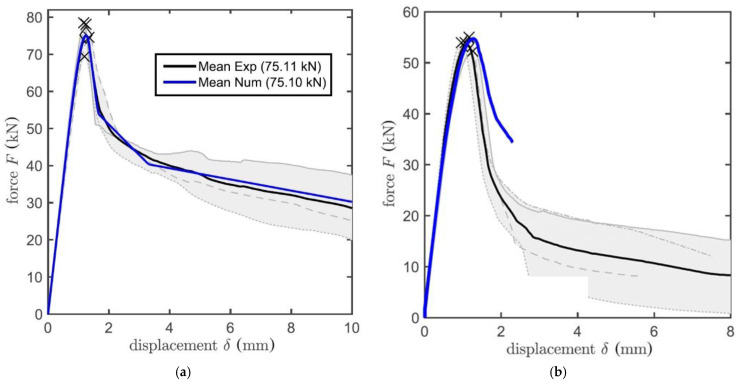
The example of post-peak behaviour of adhesive anchor—experiment (black) and the numerical model (blue); (**a**) Confined test; (**b**) Unconfined test [25].

The numerical model presented in [25] fits the post-peak behaviour of the anchor very well. The model uses the elastic response defined by the damage stiffness parameter depending on the slippage. The post-peak behaviour is defined by multilinear softening. The comparison of the numerical model and the results of the deformation-controlled confined and unconfined tests are shown in Figure 3a,b. The parameters of these anchors were: *d* = 12 mm, *h_ef_* = 90 mm, τ = 22.15 MPa and *f_c_* = 25.96 MPa.

The post-peak behaviour of the anchor in cases of concrete failure, combined concrete–bond failure and bond failure can be characterised as quasi-brittle. A large number of parameters determines the failure of the bond interface. The behaviour after the interface failure is determined by the friction effect, which mainly affects the residual strength.

## 2. Definition of Materials

Epoxy resins are often used as adhesives due to their excellent mechanical and physical properties, high adhesion, thermal stability, low shrinkage and resistance to solvents. With regard to the goal of achieving the highest possible values of mechanical properties, especially shear strength, epoxy resins were used in this analysis. In addition, specific fillers were added to the epoxies to improve their properties [26,27]. These fillers are often used because they are able to influence many physical and mechanical properties due to their structure and various surface modifications. Frequently investigated fillers are titanium dioxide [28], graphene oxide [29,30], carbon nanotubes [31,32], nanoclay [33], carbon fibres [34], etc. or even combinations thereof. The main disadvantage of common epoxy resin is the problem of curing at low-temperature conditions. The curing of standard epoxy resins does not take place at temperatures below 15 °C. In order to use the epoxy-resin-based adhesive in the construction industry, it is necessary to ensure the ability to form glued bond even during colder periods, when temperatures are between 5 and 10 °C. Processes such as gelation and vitrification are controlled by the temperature and curing time [35].

An example might be the results of standard structural epoxy adhesive (Sikadur 31). It was found that curing took place even at temperatures around 5–10 °C. However, the process was significantly slowed down. It took several days to reach an 80% conversion rate. The glass transition temperature rose even more slowly during curing at low temperatures. At 0 °C, the curing of the resin was completely inhibited [36].

### 2.1. Resins

Due to the high demands on mechanical properties, two types of resins were tested:

- Derakane 411—Vinyl ester resin;
- CHS EPOXY 531—Epoxy resin.

The CHS-EPOXY 531 resin is a low molecular weight epoxy resin modified with a bifunctional reactive solvent based on diglycidyl ether. Epoxy resin was cured with standard low molecular weight amine hardener P11. Vinyl ester resin was cured with 1.5 phr of 50% dibenzoyl peroxide paste accelerated with 0.5 phr of N, N-Dimethyl-para-toluidine.

Epoxy resin, as expected, had better mechanical properties. However, vinyl ester resins, due to their reaction mechanism, can be more easily adapted to cure at temperatures close to 0 °C.

### 2.2. Fillers

Subsequently, the effect of fillers was tested on both resins.

#### 2.2.1. Ground Limestone

Ground limestone in a fraction of 0–0.5 mm was used as a filler. This filler was especially used to reduce the significant shrinkage effect during the curing time of vinyl ester resin. However, the vinyl ester resin showed significantly worse mechanical properties in the following tests and was excluded from testing of different kind of fillers.

#### 2.2.2. Carbon Fibres

Furthermore, recycled ground carboNXT milled PURE carbon fibres with a mean fibre length of 100 µm, and a diameter of 6 µm were tested as a filler. Mixtures with different amounts of the filler were tested, particularly with 10, 30 and 50 phr.

#### 2.2.3. Nano Fillers

Nano fillers were also tested together with epoxy resin in an effort to increase mechanical properties. The following species from the category of Nano fillers were tested:

- Carbon nanotubes (Tubal Matrix 201);
- Graphene oxide (GO 21, GO 43).

Preparation of the samples with nanofillers was performed according to the manufacturer’s recommendations. The recommended Tubal Matrix 201 concentration of 0.5 phr and 0.03 and 0.06 phr for each graphene oxide was tested. A concentration of 0.5 phr for graphene oxide was also experimentally prepared for comparison.

#### 2.2.4. Curing Accelerators

Several approaches have been tried to accelerate the curing reaction with the aim to improve the curing time, even at temperatures of 5–10 °C.

First, the thiol-based hardener pentaerythritol tetrakis (3-mercaptopropionate) was tested. Then, the use of boric acid and triethyl phosphate, both of which are known for accelerating the curing reaction, were tried in mixtures with a standard amine hardener. Finally, a low-temperature hardener based on the polyfunctional amine AN2609 was tested.

All the recipes for tested mixtures are presented in Appendix A.

### 2.3. Steel and Concrete

Anchor bolts were substituted by threaded rods made of steel grade 12.9 with no surface finish. The use of steel with high strength characteristic was chosen to prevent steel anchor bolt failure during the tests. The base test specimens, as it is mentioned in the methodology description below, were made from a standard steel grade of S355. For further comparison, concrete base specimens were used, made of concrete with a cubic strength of 80.77 MPa (tested on cubes with 150 mm edge length).

## 3. Testing Methodology

With increasing demands on the reliability of the anchor system, the development of high-strength materials and the effort to expand the applicability of such anchor systems, it is still necessary to look for new, more effective contact materials (adhesives).

Due to the fact that in many cases it is necessary to verify the mechanical properties of the developed contact material with the lowest possible costs and time requirements, it is not entirely effective to use established testing methodologies that depend on the preparation of the concrete testing specimens. An example of the suitability of the described methodology can be researched in the field of using concrete with a compressive strength greater than 60 MPa together with a steel anchor bolt stronger than 800 MPa while maintaining the minimum possible anchorage lengths [9]. In such a configuration, the weakest element of the system is usually the contact material, and the resulting load-bearing capacity of the system is no longer significantly affected by the characteristics of the concrete. In such a case, it is time and economically inefficient to verify the mechanical properties of the developed contact material in the concrete test specimens.

### 3.1. Test Principle

The principle of the developed test is based on the so-called limited tensile test of the chemical anchor according to the guideline for European technical approval ETAG 001 [7], Figure 1.

The parameter entering into the calculation of the load-bearing capacity of the glued anchor according to [1,6,12] is the bond strength between the anchor bolt and the base material, which is mediated by the adhesive. The mechanism of failure may generally vary according to the stress distribution and the position and the shape of the failure. The failure can occur at both interfaces, concrete–adhesive or adhesive–anchor, or even inside the adhesive layer itself [37]. The bond strength value determined by the test according to Figure 1 may not be directly related to a specific type of failure. It is a parameter calculated from the applied force and the anchor geometry.

During the testing itself, the main monitored parameter is the force required to pull the anchor bolt out of the base body. From this force, the stress on the surface, defined by the outer edge of the thread of the anchor bolt and the length of the anchorage, is then determined. The stress corresponding to the maximum load of the anchor bolt is considered the limit value of bond strength (7).

(7) $$\tau =\frac{F}{\pi \cdot d\cdot a},$$

where *F* is the peak value of applied tension force, *d* is the diameter of the anchor bolt and *a* is the anchorage length.

**Figure 4:**
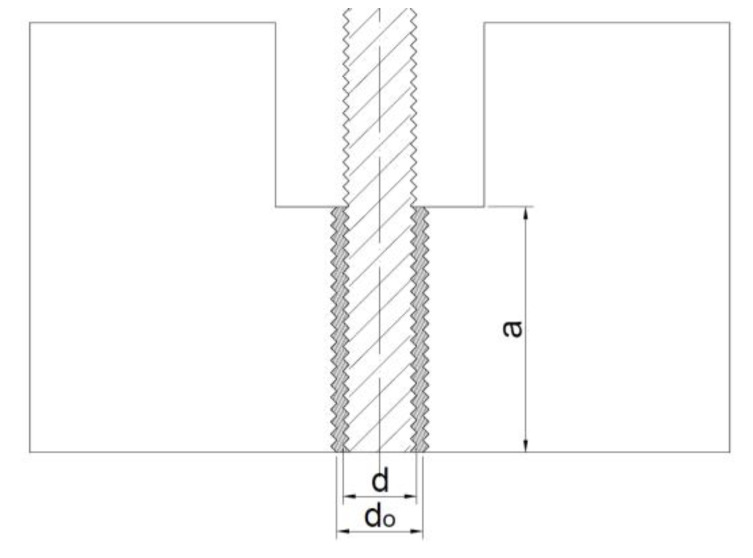
Enlarged section of an adhesive layer in steel test base specimen.

**Figure 5:**
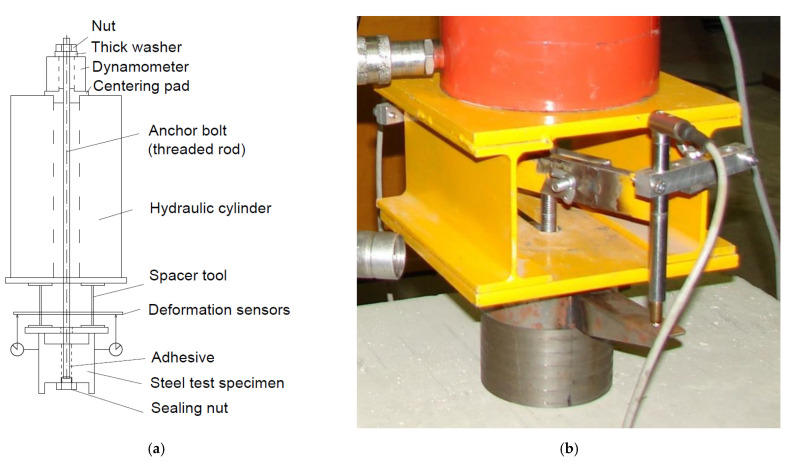
(**a**) Scheme of the test; (**b**) Detail of the deformation sensors installation.

When the adhesive failure is decisive, it occurs at the interface between the adhesive and the anchor bolt thread, which is defined by the diameter *d*. This is valid for the anchor installed in the concrete base specimen (Figure 2b) as well as for the anchor installed in the steel base specimen (Figure 4). In this modified methodology, the concrete–adhesive interface in the interface is replaced by another internal thread (diameter *d_o_*). The failure naturally occurs on a smaller area, i.e., again, on a cylindrical area defined by the outer edge of the anchor bolt thread. The scheme of the whole test system is displayed in Figure 5.

**Figure 6:**
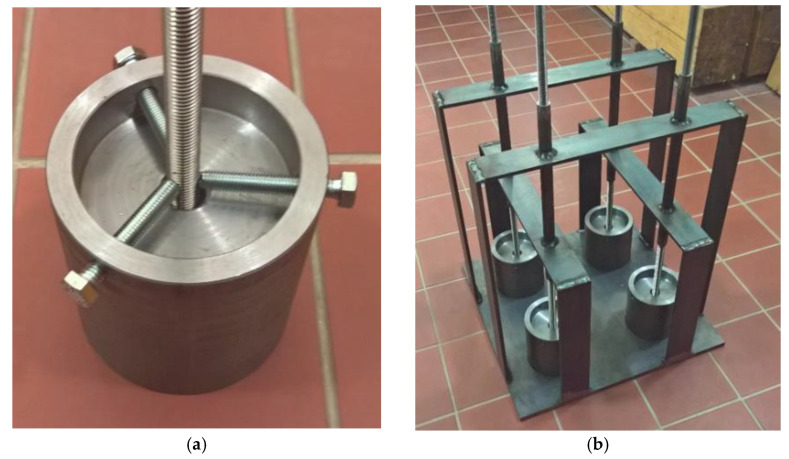
(**a**) Steel test specimen with the locking screws; (**b**) Four steel test specimens in mounting jig keeping anchor bolts in the exact vertical positions.

A new steel test specimen was designed; Figure 6. This test specimen may be made of common-strength steel. This specific steel specimen was designed to test M12 diameter bolts and was equipped with an M18 internal thread. This solution ensures an even layer of anchoring material with a thickness of at least 1.3 mm. The thickness of this layer varies depending on the change in the geometry of both threads. Due to the material properties of steel, it can be assumed that during the test, the fault always occurs in the layer of the adhesive, usually in contact with the outer edge of the thread of the anchor bolt. The steel specimen was designed to test anchor bolts anchored to a length equal to four or five times the anchor bolt diameter. The anchorage length can be regulated by exchanging the sealing screw with a different length.

To ensure the required quality of the results, it is important that the anchor bolt is installed in the ideal straight position.

This position must be ensured throughout the curing process of the adhesive. For these purposes, two options for locking the anchor bolt have been proposed. The first option is to use three locking screws, located in the upper part of the steel test specimen, which can be used to adjust the anchor bolt to a vertical position. Another option is to use a special mounting jig.

The threaded rod of strength grade 8.8, 10.9 or 12.9 can be used as the anchor bolt. Using a long nut, short anchor bolts can also be tested. The threaded rod must be properly cleaned and degreased before the test. The choice of threaded rod material is related to the quality of the tested adhesive. For high-quality adhesives, it is necessary to use steel 10.9 or 12.9 with an anchorage length of 4*d* in order to avoid failure by breaking the anchor bolt.

To measure deformations, there should be at least two displacement sensors applied between the steel specimen body and the anchor bolt. The measuring range should be at least 0–10 mm with an accuracy at least 0.001 mm. The applied force is measured by a dynamometer with an internal hole for the passage of a threaded rod. The minimum rated load of the dynamometer is 200 kN with an accuracy of 0.01 kN.

**Figure 7:**
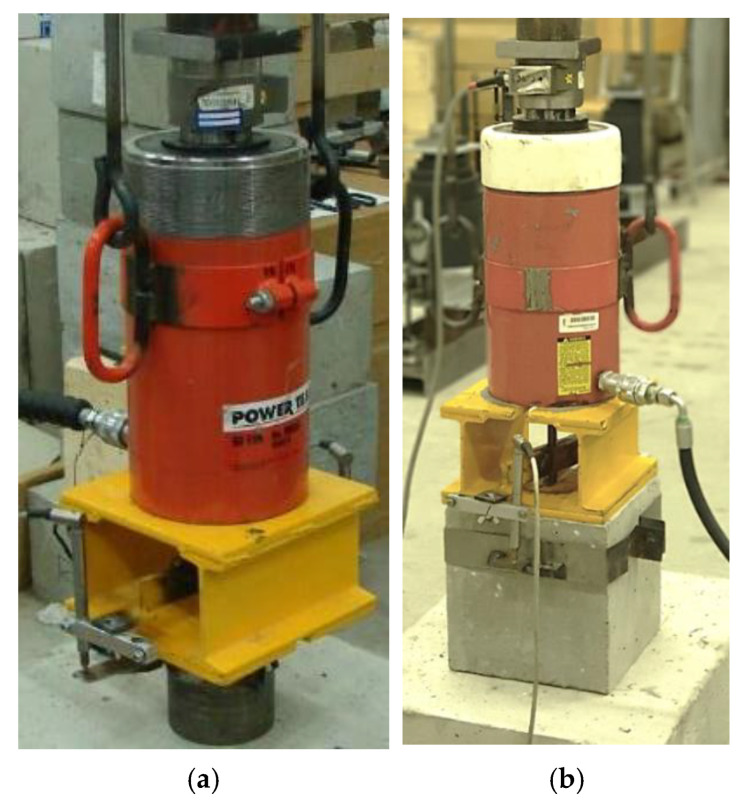
(**a**) Test with a steel base specimen; (**b**) Test with a concrete base specimen.

To derive the tensile force, it was necessary to use a hydraulic loading cylinder with an internal hole, with a load range of 200 kN; Figure 7.

#### 3.1.1. Installation of Anchor and Assembling of Loading and Measuring System

- Installation of the anchor bold in clean and dry steel specimen with an exact amount of adhesive.
- Attaching of the deformation sensors.
- Deployment of the hydraulic cylinder on the mounting plates.
- Installation of the dynamometer.
- Activation of the anchor with the nut and the mounting plate with a precise opening.

#### 3.1.2. Testing

- The measuring and sampling frequency of the data measurements should be at least 10 Hz.
- The initial load at the start of the test must not exceed 30% of the expected limit load.
- The load should be increased by constant speed until the failure occurs. The total loading time should be in the range of 40–120 s.
- A visual inspection of the anchorage system faults is performed, in particular, the integrity of the contact material for the expected anchorage length.

#### 3.1.3. Steel Test Specimen Preparation

**Figure 8:**
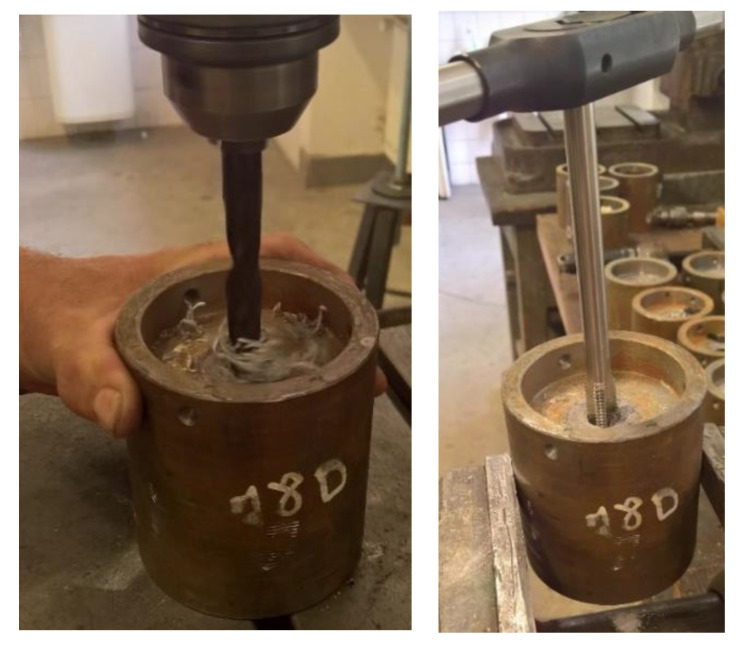
Steel base specimen restoration cleaning procedure.

Steel specimens have to be properly cleaned and degreased before the anchor bolts are installed. It is necessary to perform cleaning according to the following recommended procedure, Figure 8.

- Complete removal of the anchor bolt from the test specimen.
- Loosening and removing the sealing screw.
- Cleaning of the opening with the drill of a diameter smaller than the diameter of the inner thread.
- Cleaning of the inner thread by the usage of an appropriate screw tap.
- Cleaning the seating plate with an opening for the sealing screw.
- For a new application, the sealing screw must be re-inserted, preferably by using a sealing Teflon tape, to prevent the adhesive from penetrating the inner thread.
- Due to the wear occurring during cleaning, the number of uses of the test specimen is limited to a maximum of 10 tests. To monitor the number of uses, suitable test fixtures are properly identified by a serial number or other code.

#### 3.1.4. Test Evaluation

The result of each test is the load-deformation diagram of the adhesive, i.e., the dependence of the applied tensile force on the vertical deformation of the anchor bolt. The relationship between the applied force and the stress on the contact between the anchor bolt and the tested sample of anchor material can be described by relation (7).

Results where the working diagram is discontinuous or where the course does not have the character of bilinear behaviour with strain hardening should be excluded from evaluation. Furthermore, it is necessary to exclude such test results where an otherwise unexpected course of the test or an unexpected form of sample failure has occurred.

## 4. Experimental Analysis

### 4.1. Bond Strength Tests

**Figure 9:**
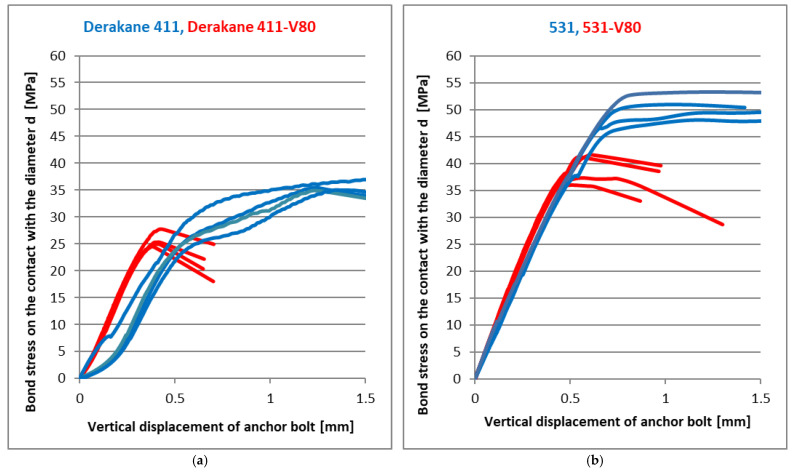
The effect of 80% of grounded limestone (V80) in the mixture: (**a**) Deracane 411; (**b**) CHS Epoxy 531 [9].

**Figure 10:**
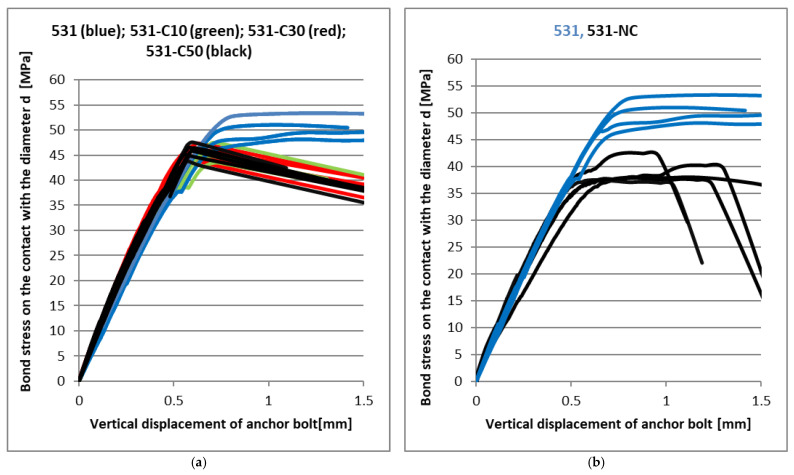
(**a**) CHS Epoxy 531 without carbon fibres; (**b**) CHS Epoxy 531 with nanotubes [9].

**Figure 11:**
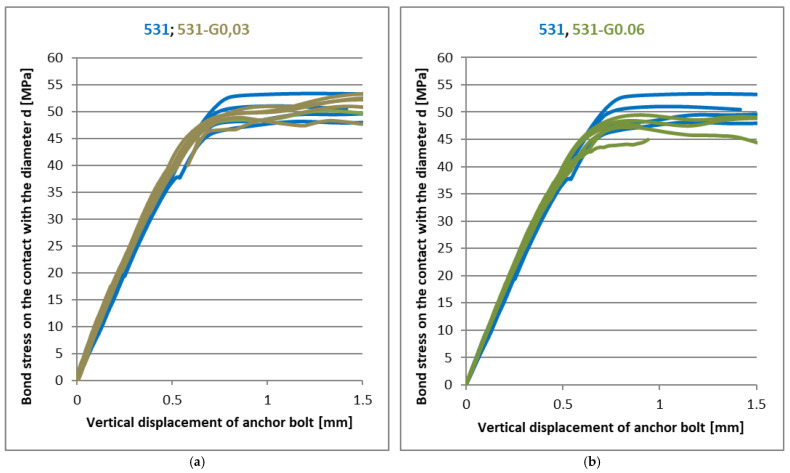
The effect of the graphene oxide GO21 on CHS Epoxy 531 (**a**) 0.03 phr; (**b**) 0.06 phr.

**Figure 12:**
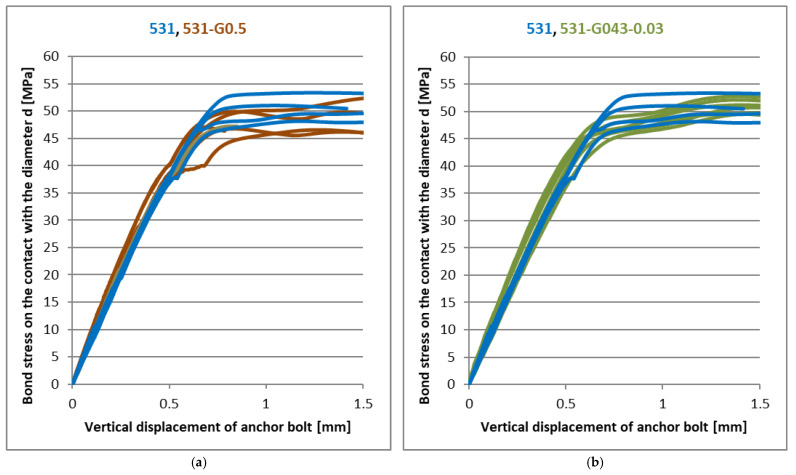
(**a**) The effect of the graphene oxide on CHS Epoxy 531 (**a**) GO21—0.5 phr; (**b**) GO43—0.03 phr.

**Figure 13:**
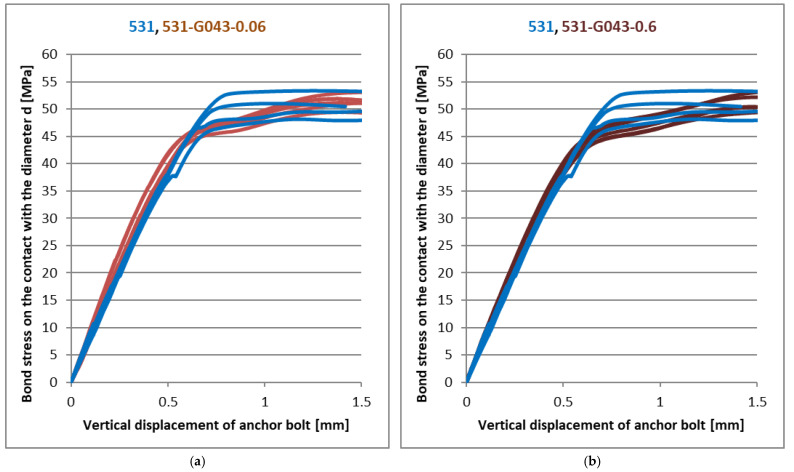
The effect of the graphene oxide GO43 on CHS Epoxy 531 (**a**) 0.06 phr; (**b**) 0.6 phr.

The following graphs show the load-deformation diagrams of the tested mixtures of the resins. The bond strength evaluated from the applied load according to relation (7) is on the vertical axis. The appropriate vertical deformation measured on the anchor bolt is displayed on the horizontal axis. The load was applied by the rising axial force. There is a small part of the load-deformation curve left in each load-deformation diagram in the following figures to show the peak value clearly, but it does not describe the post-peak behaviour and the residual strength very well. Figure 9 shows the results for basic Derakane 411 and 531 mixtures in comparison with the same resin with a marginal amount (cca 80%) of milled limestone as a filler. It is obvious that the shrinkage effect of vinyl-ester resin is reduced together with the significant reduction of the final adhesive strength, as expected. At the same time, however, there was an increase in the stiffness of the joint, which is best seen in Figure 9a, where a significant reduction in vertical displacement with a significant linear dependence can be seen, and consequent brittle failure occurred. As can be seen from Figure 9a, the bond strength of the Derakane 411 mixture does not reach 35 MPa, but the failure already occurs at a stress of about 25 MPa. From this point of view, the addition of mineral filler significantly increased the stiffness of the joint, and at the same time, there was only a slight decrease in the bond strength. In contrast to the Derakane 411 mixture, the 531 mixture produces significantly less shrinkage and probably less cracking during curing. From the dependence of the joint strength and the vertical displacement in Figure 9b, it is clear that the addition of the mineral filler increased the joint stiffness only slightly, but the joint strength decreased by about 25%. Following Figure 10, Figure 11, Figure 12 and Figure 13 show the effect of different types (carbon fibres, nanotubes, graphene) and the amounts of these fillers on the adhesive strength characteristics. Figure 9a shows the effect of the addition of milled carbon fibres in several concentrations. There was a slight improvement in the linear part of the load-deformation dependence, which may be caused by the bridging of microcracks, which arise during curing or during loading, but the ultimate strength decreased by about 10%. The reason for the reduction in ultimate strength is not clear from these tests. However, the addition of ground carbon fibres significantly increased the viscosity of the adhesive, which could adversely affect some parameters, such as the quality of surface wetting, the formation of air pores during mixing, etc.

The behaviour of the mixture with the addition of nanotubes in Figure 10b proved to be completely unexpected. There was only decreasing in the ultimate strength by about 25%. It is possible that nanotubes are not suitable for this type of use, or an unsuitable method of mixture preparation was chosen.

Figure 11, Figure 12 and Figure 13 show the results of experiments with another type of nanofiller. Graphene oxide was used in two modifications (GO21 and GO43), differing in the way its surface was oxidised. Each of these modifications was tested at three different concentrations. It is clear from Figure 11, Figure 12 and Figure 13 that the addition of these nanofillers had a positive effect, especially at concentrations of 0.03 and 0.06 phr, when there was a slight improvement in the linear part of the load curves and the decrease in ultimate strength was in percentage units at the same time. With a graphene oxide content of 0.5 resp. 0.6 phr, the improvement in the linear part of the load curve was not so significant.

### 4.2. Curing Tests at Low Temperatures

**Figure 14:**
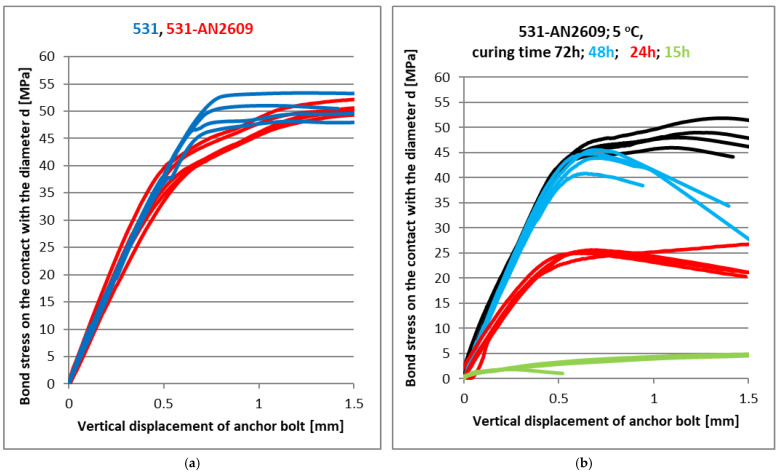
CHS Epoxy 531 with AN2609 curing accelerator; (**a**) Curing at 20 °C; (**b**) Curing at 5 °C.

**Figure 15:**
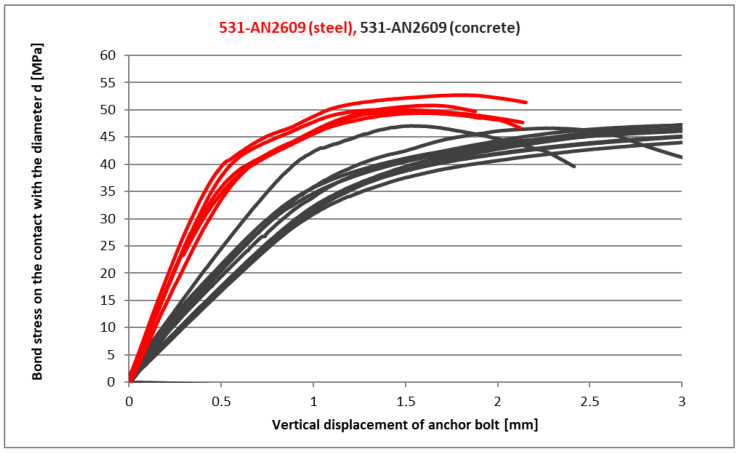
A comparison of CHS Epoxy with accelerator AN2609 in concrete and steel base specimens.

In these tests, the base mixture of the epoxy resin 531 with the curing accelerators was verified. The polyfunctional amine AN2609 has proven to be a suitable hardening accelerator to reduce the low-temperature hardening time. Figure 14a shows the bond strength test at a normal temperature as a comparison with the basic 531 mixture. This type of mixture was also tested by the standard confined test according to ETAG [7] in high-strength concrete (with a compressive strength higher than 87 MPa). Figure 15 presents a comparison of the confined test in concrete base bodies and the presented simplified tests in steel base bodies. It can be seen that the resulting maximum strength values according to relation (7) are comparable for both tests. The main difference in the load-deformation diagram is the stiffness of the anchor installed in the concrete base specimen. It only reaches half of the value for the anchor in the steel base specimen.

**Figure 16:**
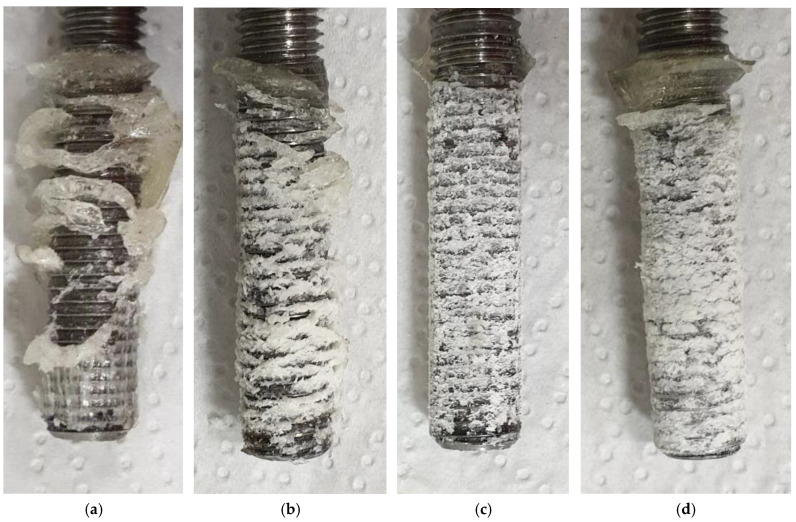
Failure of 531-AN2609 at 5 °C, curing time: (**a**) 15 h; (**b**) 24 h; (**c**) 48 h; (**d**) 72 h.

**Figure 17:**
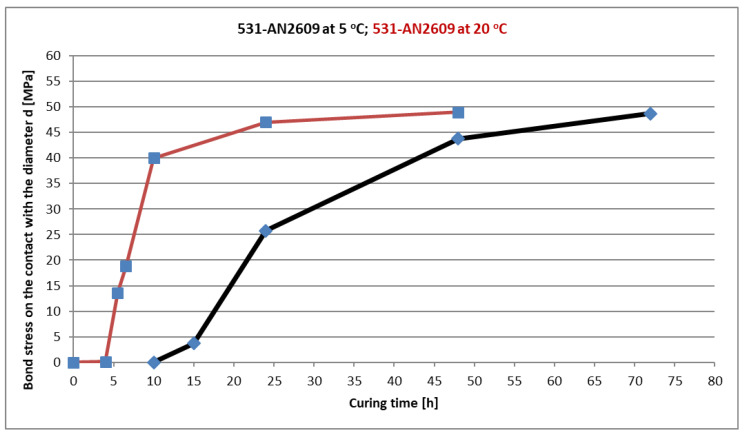
CHS Epoxy 531 with AN2609 curing accelerator. Bond strength is dependent on the curing time at a temperature of 5 °C and room temperature of 20 °C.

All steel test specimens, adhesive compounds and anchor bolts were first tempered in a climate chamber to a temperature of 5 °C for further use in low-temperature curing tests. Altogether, 16 tests were performed, with four individual tests after 15, 24, 48 and 72 h of curing. The results of these tests are shown in Figure 14b. The specimens after the failure for selected curing time intervals are presented in Figure 16. The adhesive still has almost the same plastic state after 15 h of curing at a temperature of 5 °C; however, it already reached some initial strength. Then, after 24 h, the behaviour of the adhesive already reached the standard load-deformation diagram with some linear part. The peak value of the bond strength reached 50 % of the final bond strength value. The curing time of 48 h was sufficient to reach 80 % of the final bond strength value, which was reached after 72 h of curing. Figure 17 shows the dependence of the bond strength on the curing time of this mixture at a temperature of 5 °C in comparison to 20 °C. The curing time for the final strength at 5 °C was three times longer than for a room temperature of 20 °C. Strength equal to 50 % of the final value was reached at 5 °C after a time period that was 2.4 times longer than the curing time for 20 °C.

### 4.3. Long-Term Loading Tests

**Figure 18:**
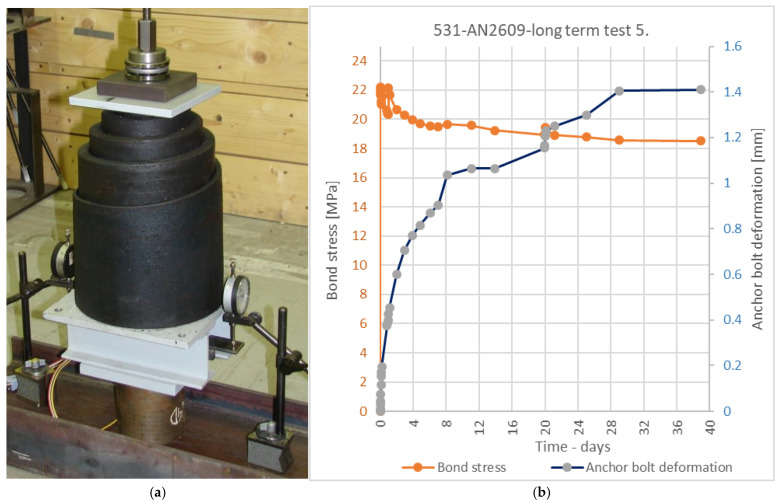
(**a**) Long-term test CHS Epoxy 531 with AN2609 curing accelerator; (**b**) One long-term test result.

**Figure 19:**
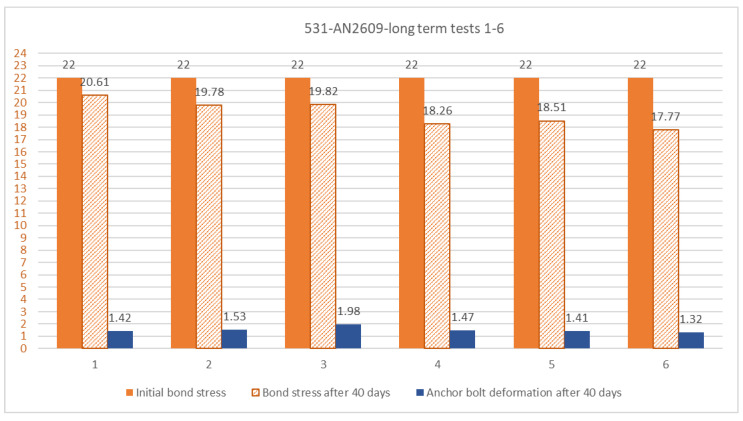
Long-term test summarization.

To prove the stability of the fully cured mixture, a long-term loading test was also performed on the 531-AN2609 mixture. The principle of the test is similar to the one shown in Figure 5, but the hydraulic cylinder is substituted with a conical train spring and the load is applied manually by tightening the top nut on the ball-bearing. The load was applied 24 h after the anchor installation. The adhesive was cured at a temperature of 24 °C. At the time of the application of the load, the adhesive reached strength values higher than 95% of its final strength. The load level for the long-term test was determined on the basis of the ETAG recommendation [7], to the load equal to 50 % of the characteristic strength value determined from static tests. The characteristic value of bond strength of the 531-AN2609 sample was determined as 39.4 MPa (using the recommended coefficient of variation 2.57 [7]). Therefore, the level of the initial load was set to imply a bond stress of 19.7 MPa. The test proved that it is very difficult to maintain this level of load. The rising deformation of the bonded anchor causes the release of the conical spring and thus a significant reduction in the load. To compensate for this effect, a procedure was chosen in which a force 15% higher than the required value was applied during the initial load. Then, the applied force was again raised in two steps after 1 h and after 24 h from the beginning of the loading. The test result presented in Figure 18b shows that the applied force stabilises after 1 month of the load duration, together with the stabilisation of the deformation of the bonded anchor, which, after this time period, is more than seven times higher than the deformation of the same specimen in the short-term test at the same load level. Together, six tests were performed. The result after 40 days of load duration is shown in Figure 19.

## 5. Summarization of Bond Stress Results

**Figure 20:**
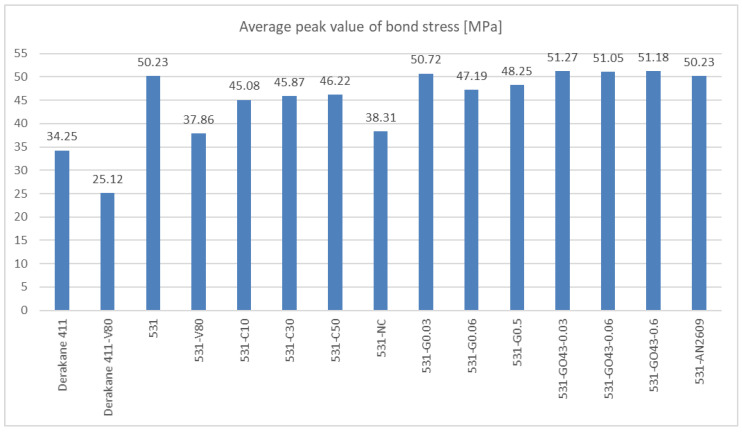
A comparison of average value of peak bond strength of the tested mixtures.

The results of experiments focused on the bond strength and type of fillers are summarised in the following Figure 20. All results were obtained by the presented simplified testing method using the steel base specimens.

Figure 20 shows the comparison of peak values of bond stress evaluated as an average peak value from individual tests for each mixture. As it is evident, the effect of the filler in the form of carbon fibres, nanotubes or graphene oxides is limited in combination with the used epoxy resin.

**Figure 21:**
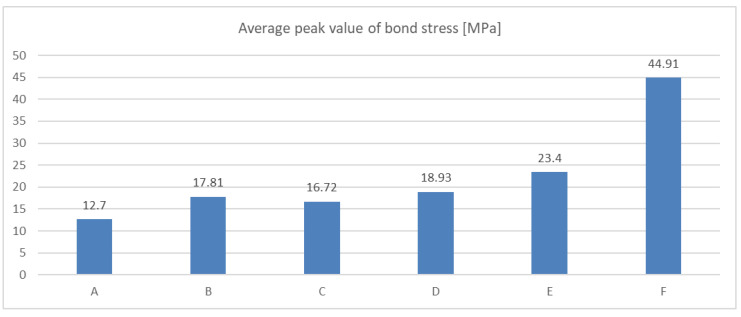
Comparison of bond strength of selected adhesive types: (A)—Average value of large number of adhesives [39], (B)—Hilti-HVU-C20/25 [38], (C)—Hilti-R150-C20/25 [38], (D)—Hilti-RE500-C20/25 [38], (E)—Hilti-RE500-C40/45 [38], (F)—531-AN2609-C65/70.

Figure 21 shows the peak bond stress of several common industrial adhesives used for anchoring [38] in comparison with the final presented product 531-AN2609. There is also an average value of a larger group of adhesives and mortars test results published in [39]. It is obvious that the concrete strength influences the final bond strength value. According to the ETAG standard [7], it is possible to normalise these results to concrete C20/25; however, that is possible only when the failure occurs on the interface between the concrete and the adhesive.

**Figure 22:**
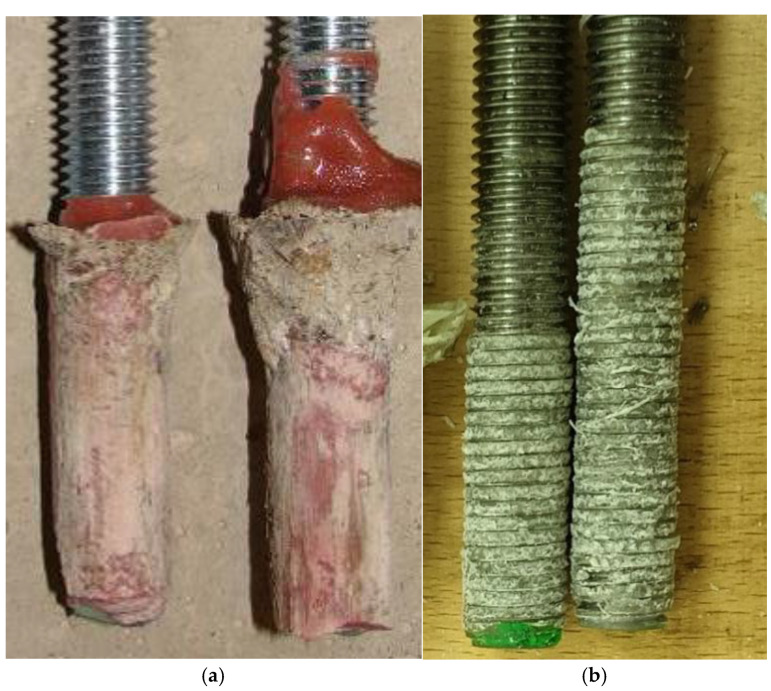
Failure position—(**a**) HILTI RE500 in concrete C20/25; (**b**) 531-AN2609 in concrete C65/70.

Results presented in Figure 21 are not fully comparable in this way. The failure for mixture 531-AN2609, which was tested in concrete C65/70, occurred on the interface between the steel bolt and the adhesive. The difference in the failure position is visible in Figure 22, which shows a comparison of two different specimens after the test. The fact that the failure in the test with high-strength concrete occurred on the interface between the adhesive and steel bolt is the main vindication for the presented simplified testing method to be more economical and suitable for the development of new adhesives.

### Cost Comparison and Recommendations for Engineering Practice

The above-mentioned groups of fillers were selected to verify the effect of their content on the strength of the adhesive and to find the optimal price–performance ratio. The properties and cost of adhesive are major parameters in engineering practice. The manufacturers of adhesives have to balance the optimal value of the price in respect to the strength characteristics and other properties.

Fillers like ground limestone are used to reduce the price of the adhesives, often in very high concentrations. It seems advantageous to consider replacing a substantial volume of adhesive with a cheap filler. They have some other benefits, such as reduced shrinkage or increased compressive strength, but most other properties deteriorate. Limestone-type fillers are 25–50 times cheaper than the binder per unit weight, respectively 10–20 times cheaper per unit volume, but a high concentration of fillers can reduce the processability of the adhesive, which can lead to problems in its practical application. The amount of the filler has to be in balance with the final bond strength, which will be decreased. For example, for mixture 531-V80 in comparison with the basic mixture 531, the peak value of bond stress decreased by 25 %, but the cost per unit volume of adhesive decreased by 55%.

On the other hand, fillers like ground carbon fibres or nanofillers are more expensive per unit volume than a binder.

Milled recycled carbon fibres are at least twice as expensive as a binder, but can be up to 10 times more expensive if non-recycled material is used. The high concentration of these fillers also radically reduces the processability of the adhesive, which can lead to problems in its practical use. It has been shown that improper selection of the type and amount of filler can adversely affect both the final value of cohesion and other useful properties of the adhesive, such as viscosity, surface-wetting quality, etc. These phenomena can have a negative effect on the adhesive production process. Inhomogeneity, uneven mixing of the filler, formation of fibre clumps and/or cracking and air pores can occur. These fillers have also been used in high concentrations, resulting in an increase in adhesive costs. However, an increase in price is justified only if there is a corresponding improvement in mechanical properties. The positive economic impact of using fillers to improve the properties of the epoxy resin for anchoring appears to be sporadic. It may be advantageous to use very cheap waste carbon fibres in a reasonable concentration to improve the linear part of the working diagram and thus increase the characteristic value of the bond strength.

Nanofillers are also more expensive per unit volume than a binder is. In the case of graphene oxide, which is still a development product, the price level has not been determined, but nanotubes are 300–700 times more expensive than a binder is. Fortunately, nanofillers are added in very small amounts, so that the final price increases by about 10–30%. Everything that has been said about the effect of the addition of ground carbon fibres on the properties of the binder also applies in the case of nanotubes. However, even more attention needs to be paid to the dilution procedure and uniform distribution of the filler in the resin in the case of the processing of nanofillers, because it plays a key role in enhancing the properties of the final compound.

## 6. Conclusions

One of the main parts of the presented analysis is the development of the simplified method for verifying the shear strength characteristics of the adhesive. In this method, steel base specimens with internal threads are used instead of concrete. These specimens can be cleaned repeatedly and reused. Although the method cannot describe the exact behaviour of the interface between the adhesive and the concrete, it describes with sufficient accuracy the main parameter that affects the resulting connection with the concrete, and that is the shear strength of the adhesive. For this reason, it is very suitable for the quick verification of changes in the strength characteristics of adhesives during their development. It is extremely suitable for testing the effect of fillers, which do not have a significant effect on the chemical bond with the concrete, but aim to improve the useful properties of the adhesive.

### 6.1. Bond Strenght Test Conclusion

The study presented in this paper was focused on the bond strength testing of adhesives suitable for use in combination with high-strength concrete and high-grade steel. Epoxy and vinyl ester resins were used in the tests, which were chosen for their very good mechanical properties. As expected, epoxy resins had better mechanical properties and are best suited for combination with other high-performance materials. Further, the use of various fillers was tested in an effort to improve mechanical properties. The main findings are:

- The results presented in this analysis show that the use of fillers in the form of carbon fibres, nanotubes or other types of nano-fillers might not lead to a resulting improvement in the mechanical properties of the epoxy adhesives. It is possible to prepare a pure epoxy resin with a very high value of resulting strength. The use of fillers of these types does not bring any other benefits, such as a reduction in the price of the final product. It has been shown that the improper selection of the type and amount of filler can adversely affect both the final value of cohesion and other useful properties of the adhesive, such as viscosity, surface wetting quality, etc. These phenomena can have a negative effect on the adhesive production process. Inhomogeneity, uneven mixing of the filler, formation of fibre clumps and/or cracking and air pores can occur. The high concentration of fillers also radically reduces the processability of the adhesive, which can lead to problems in its practical application.
- Of the tested fillers, only ground limestone appears to indicate a positive effect in the reduction of the shrinkage of the vinyl ester resin. This filler can be used in very high concentrations, which, thanks to its price, brings significant cost savings together with an acceptable reduction in the resulting mechanical properties.

### 6.2. Low-Temperature Test Conclusion

Another part of this analysis was focused on the possibility of shortening the curing time at low temperatures. This problem is very significant, especially for epoxy resin. First, the thiol-based hardener pentaerythritol tetrakis (3-mercaptopropionate) was tested. Then, the use of boric acid and triethyl phosphate, which are both known for the accelerating of the curing reaction, were tried in mixtures with standard amine hardener. The addition of both substances to the system significantly accelerated the reaction.

- Boric acid shows higher reactivity, even at lower concentrations;
- Triethyl phosphate has an overall lower potency, but it is also able to accelerate the curing reaction.

A general problem with the preparation of an adhesive suitable for low temperatures is the requirement of maintaining a sufficient time before the start of the reaction, necessary for practical use at common temperatures. The final product, 531-AN2609, managed to balance these requirements. The result is an adhesive that can be used both at common temperatures (20 °C) and at a temperature close to zero degrees Celsius (5 °C). The curing time for low temperatures is about three times the curing time at common temperatures.

### 6.3. Future Research

This article has focused mainly on how to measure the strength of adhesives for anchors, but there are still many unresolved issues in the field of binder formulation and curing of the adhesives. Future research can be focused on the individual components of adhesive, accelerators ad fillers, their type and amount and particle size synergistic effects. Since the adhesives are designed for low curing temperatures, a large area for further research is the study of additional curing as the temperature rises to normal service temperatures and the effect of the curing and adhesive formulation to an achievable glass transition temperature and thus the maximum service temperature.

## Data Availability

The data underlying this study will be available on reasonable request to the corresponding author.

## Appendix A

The following tables represent all tested recipes of the resins.

**Table A1:** CHS Epoxy 531 [9].

Characteristics	CHS-EPOXY 531
Dynamic viscosity. 25 °C (MPa·s)	1500
Tensile strength (MPa)	50
Bending strength (MPa)	90
Shrinkage (%)	0.3
Epoxy equivalent (g·mol^−1^)	175–182

**Table A2:** Derakane 411 vinyl ester resin [9].

Characteristics	Derakane 411
Dynamic viscosity. 25 °C (MPa·s)	430
Styren (%)	33
Tensile strength (MPa)	90
Tension modulus (GPa)	3.3
Bending strength (MPa)	145
Bending modulus (GPa)	3.4
Shrinkage (%)	7.5
Glass transition temperature (°C)	135

**Table A3:** The basic recipe of CHS Epoxy 531 [9].

Mixture	Compound	Weight (g)
531	CHS-Epoxy 531	100
	P11	12

**Table A4:** The basic recipe of Derakane 411 [9].

Mixture	Compound	Weight (g)
Derakane 411	Derakane 411	100
	DBP	1.5
	DMPT	0.5

**Table A5:** Grounded limestone filler in CHS Epoxy 531 [9].

Mixture	Compound	Weight (g)
531-V80	CHS-Epoxy 531	100
	P11	12
	ground limestone	400

**Table A6:** Grounded limestone filler in Derakane 411 [9].

Mixture	Compound	Weight (g)
Derakane 411-V80	Derakane 411	100
	DBP	1.5
	DMPT	0.5
	ground limestone	400

**Table A7:** Carbon fibres in CHS Epoxy 531, 10 phr [9].

Mixture	Compound	Weight (g)
531-C10	CHS-Epoxy 531	100
	P11	12
	Milled carbon fibres	10

**Table A8:** Carbon fibres in CHS Epoxy 531, 30 phr [9].

Mixture	Compound	Weight (g)
531-C30	CHS-Epoxy 531	100
	P11	12
	Milled carbon fibres	30

**Table A9:** Carbon fibres in CHS Epoxy 531, 50 phr [9].

Mixture	Compound	Weight (g)
531-C50	CHS-Epoxy 531	100
	P11	12
	Milled carbon fibres	50

**Table A10:** Nanotubes in CHS Epoxy 531-NC [9].

Mixture	Compound	Weight (g)
531-NC	CHS EPOXY 531	100.00
	Tubal matrix 201	0.5
	P11	11.00

**Table A11:** Graphene oxide GO21 CHS Epoxy 531, 0.03 phr.

Mixture	Compound	Weight (g)
531-G0.03	CHS EPOXY 531	100.00
	GO21	0.03
	P11	11.00
	Disperbyk-2152	0.03
	BYK-066N	1.10

**Table A12:** Graphene oxide GO21 CHS Epoxy 531, 0.06 phr.

Mixture	Compound	Weight (g)
531-G0.06	CHS EPOXY 531	100.00
	GO21	0.06
	P11	11.00
	Disperbyk-2152	0.06
	BYK-066N	1.10

**Table A13:** Graphene oxide GO21 CHS Epoxy 531, 0.5 phr.

Mixture	Compound	Weight (g)
531-G0.5	CHS EPOXY 531	100.00
	GO21	0.5
	P11	11.00
	Disperbyk-2152	0.5
	BYK-066N	1.10

**Table A14:** Graphene oxide GO43 CHS Epoxy 531, 0.03 phr.

Mixture	Compound	Weight (g)
531-GO043-0.03	CHS EPOXY 531	100.00
	GO43	0.03
	P11	11.00
	Disperbyk-2152	0.03
	BYK-066N	1.10

**Table A15:** Graphene oxide GO43 CHS Epoxy 531, 0.06 phr.

Mixture	Compound	Weight (g)
531-GO043-0.06	CHS EPOXY 531	100.00
	GO43	0.06
	P11	11.00
	Disperbyk-2152	0.06
	BYK-066N	1.10

**Table A16:** Graphene oxide GO21 CHS Epoxy 531, 0.6 phr.

Mixture	Compound	Weight (g)
531-GO043-0.5	CHS EPOXY 531	100.00
	GO43	0.5
	P11	11.00
	Disperbyk-2152	0.5
	BYK-066N	1.10

**Table A17:** CHS Epoxy 531 with AN2609 accelerator.

Mixture	Compound	Weight (g)
531-AN2609	CHS EPOXY 531	100.00
	AN2609	21.00
	P11	6.00
	Benzyl alcohol	7.00
	Triethyl phosphate	5.40
	boric acid	1.80
	2.4.6-tris(dimethylaminomethyl)fenol	1.80

## References

[1] Cook R.A., Kunz J., Fuchs W., Konz R.C. (1998). Behavior and design of single adhesive anchors under tensile load in uncracked concrete. ACI Struct. J..

[2] Epackachi S., Esmaili O., Mirghaderi S.R., Behbahani A.A.T. (2015). Behavior of adhesive bonded anchors under tension and shear loads. J. Constr. Steel Res..

[3] Ozbolt J., Eligehausen R., Periskic G., Mayer U. (2007). 3D FE analysis of anchor bolts with large embedment depths. Eng. Fract. Mech..

[4] Ahmed L.T., Braimah A. (2019). Tensile behaviour of adhesive anchors under different strain rates. Eng. Struct..

[5] Cook R.A., Doerr G.T., Klingner R.E. (1993). Bond stress model for design of adhesive anchors. ACI Struct. J..

[6] Ceroni F., Di Ludovico M. (2020). Traditional and innovative systems for injected anchors in masonry elements: Experimental behavior and theoretical formulations. Constr. Build. Mater..

[7] ETAG 001 (2013). Guideline for European Technical Approval of Mteal Anchors for Use in Concrete. Annex A Details Tests.

[8] Fuchs W., Hofmann J., Hulder G. (2015). Load bearing behavior of chemical fasteners after installation at decreased temperatures. Beton Stahlbetonbau.

[9] Barnat J., Bajer M., Vild M. To the Problems of Anchoring Adhesives in High Performance Concrete. Proceedings of the 3rd World Multidisciplinary Civil Engineering, Architecture, Urban Planning Symposium (WMCAUS).

[10] Eligehausen R., Mallée R., Silva J.F., John R., Eligehausen R., Mallée J., Silva F. (2006). Anchorage in Concrete Construction.

[11] Meszaros J., Eligehausen R. (1998). Einfluss der Bohrlochreinigung und von feuchtem Beton auf das Tragvehalten von Injektions-dübeln (Influence of Hole Cleaning and of Humid Concrete on the Load-Bearing Behaviour of Injection Anchors).

[12] Spieth H.A., Eligehausen R. (2002). Bewehrungsnschlüsse mit Nachträglich Eingemörtelten Bewehrungstaben (Starter Bars with Post Installed Rebars). Beton und Stahlebetonbau 97.

[13] Eligehausen R., Cook R.A., Appl J. (2006). Behavior and design of adhesive bonded anchors. ACI Struct. J..

[14] Fuchs W., Eligehausen R., Breen J. (1995). Concrete capacity design (CCD) approach for fastening to concrete. ACI Struct. J..

[15] ACI 355.4-11 (2011). Qualification of Post-Installed Adhesive Anchors in Concrete and Commentary.

[16] EN 1992-4:2018 (2018). Eurocode 2—Design of Concrete Structures—Part 4: Design of Fastenings for Use in Concrete.

[17] Cook R.A. (1993). Behaviour of chemically bonded acnhors. J. Struct. Eng. ASCE.

[18] Cook R.A., Fagundo F.E., Biller M.H., Richardson D.E. (1991). Tensile Behaviour and Design of Single Adhesive Anchors. Structure Material Research Report No. 91-3.

[19] Bajer M., Barnat J. (2012). The glue-concrete interface of bonded anchors. Constr. Build. Mater..

[20] Randl N., Gusella O. (2011). Behavior of adhesive anchors in high strength and ultra high performance concrete. Beton Stahlbetonbau.

[21] Cook R.A., Konz R.C. (2001). Factors influencing bond strength of adhesive anchors. ACI Struct. J..

[22] Ceroni F., Bonati A., Galimberti V., Occhiuzzi A. (2018). Effects of Environmental Conditioning on the Bond Behavior of FRP and FRCM Systems Applied to Concrete Elements. J. Eng. Mech..

[23] De Domenico D., Urso S., Borsellino C., Spinella N., Recupero A. (2020). Bond behavior and ultimate capacity of notched concrete beams with externally-bonded FRP and PBO-FRCM systems under different environmental conditions. Constr. Build. Mater..

[24] Lahouar M.A., Caron J.F., Pinoteau N., Forêt G., Benzarti K. (2017). Mechanical behavior of adhesive anchors under high temperature exposure: Experimental investigation. Int. J. Adhes. Adhes..

[25] Marcon M., Vorel J., Ninčević K., Wan-Wendner R. (2017). Modeling Adhesive Anchors in a Discrete Element Framework. Materials.

[26] Hou W.X., Gao Y., Wang J., Blackwood D.J., Teo S. (2020). Recent advances and future perspectives for graphene oxide reinforced epoxy resins. Mater. Today Commun..

[27] Frigione M., Lettieri M. (2020). Recent Advances and Trends of Nanofilled/Nanostructured Epoxies. Materials.

[28] Kavimani V., Prakash K.S., Thankachan T., Udayakumar R. (2020). Synergistic improvement of epoxy derived polymer composites reinforced with Graphene Oxide (GO) plus Titanium di oxide(TiO_2_). Compos. Part B Eng..

[29] Xie Y.K., Liu C.H., Liu W.Q., Liang L.Y., Wang S., Zhang F.Y., Shi H.Y., Yang M.P. (2020). A novel approach to fabricate polyacrylate modified graphene oxide for improving the corrosion resistance of epoxy coatings. Colloids Surf. A Physicochem. Eng. Asp..

[30] Wolk A., Rosenthal M., Weiss J., Voigt M., Wesendahl J.N., Hartmann M., Grundmeier G., Wilhelm R., Meschut G., Tiemann M. (2018). Graphene oxide as flexibilizer for epoxy amine resins. Prog. Org. Coat..

[31] Zamal H.H., Barba D., Aissa B., Haddad E., Rosei F. (2021). Failure analysis of self-healing epoxy resins using microencapsulated 5E2N and carbon nanotubes. Smart Mater. Struct..

[32] Wang E.L., Dong Y.B., Islam M.Z., Yu L.M., Liu F.Y., Chen S.J., Qi X.M., Zhu Y.F., Fu Y.Q., Xu Z.H. (2019). Effect of graphene oxide-carbon nanotube hybrid filler on the mechanical property and thermal response speed of shape memory epoxy composites. Compos. Sci. Technol..

[33] Wang M., Fan X.S., Thitsartarn W., He C.B. (2015). Rheological and mechanical properties of epoxy/clay nanocomposites with enhanced tensile and fracture toughnesses. Polymer.

[34] Basri A.B.A., Chae D.W., Lee H. (2020). Investigation of the dynamic characteristics of a carbon -fiber -reinforced epoxy with adhesive -jointed structure. Compos. Struct..

[35] Moussa O., Vassilopoulos A.P., de Castro J., Keller T. (2012). Early-age tensile properties of structural epoxy adhesives subjected to low-temperature curing. Int. J. Adhes. Adhes..

[36] Moussa O., Vassilopoulos A.P., Keller T. (2012). Effects of low-temperature curing on physical behavior of cold-curing epoxy adhesives in bridge construction. Int. J. Adhes. Adhes..

[37] Satoh A., Takeda K., Murakami K. (2019). FEM analysis on combined bond-cone fracture of a post-installed adhesive anchor filled with UHPFRC. Theor. Appl. Fract. Mech..

[38] Barnat J., Bajer M. (2011). Analysis of Bonded Anchor in Combined Concrete-Bond Failure Mode. Recent Research in Geography Geology, Energy, Environment and Biomedicine, Proceedings of the 4th WSEAS International Conference on Engineering Mechanics, Structures, Corfu Island, Greece, 14–16 July 2011.

[39] Cook R.A., Bishop M.C., Hagedoorn H.S., Sikes D., Richardson D.S., Adams T.L., De Zee C.T. (1994). Adhesive Bonded Anchors: Bond Properties and Effects of in Service and Installation Conditions.

